# NEMA image quality phantom measurements and attenuation correction in integrated PET/MR hybrid imaging

**DOI:** 10.1186/s40658-015-0122-3

**Published:** 2015-08-20

**Authors:** Susanne Ziegler, Bjoern W. Jakoby, Harald Braun, Daniel H. Paulus, Harald H. Quick

**Affiliations:** Institute of Medical Physics, University of Erlangen-Nuremberg, Henkestraße 91, 91052 Erlangen, Germany; Siemens Healthcare, Erlangen, Germany; University of Surrey, Guildford, Surrey UK; Erwin L. Hahn Institute for Magnetic Resonance Imaging, University of Duisburg-Essen, Essen, Germany; High Field and Hybrid MR Imaging, University Hospital Essen, Essen, Germany

**Keywords:** NEMA image quality measurements, Integrated PET/MR hybrid imaging, PET/MR phantom measurements, CT-based attenuation correction, MR-based attenuation correction

## Abstract

**Background:**

In integrated PET/MR hybrid imaging the evaluation of PET performance characteristics according to the NEMA standard NU 2–2007 is challenging because of incomplete MR-based attenuation correction (AC) for phantom imaging. In this study, a strategy for CT-based AC of the NEMA image quality (IQ) phantom is assessed. The method is systematically evaluated in NEMA IQ phantom measurements on an integrated PET/MR system.

**Methods:**

NEMA IQ measurements were performed on the integrated 3.0 Tesla PET/MR hybrid system (Biograph mMR, Siemens Healthcare). AC of the NEMA IQ phantom was realized by an MR-based and by a CT-based method. The suggested CT-based AC uses a template μ-map of the NEMA IQ phantom and a phantom holder for exact repositioning of the phantom on the systems patient table. The PET image quality parameters contrast recovery, background variability, and signal-to-noise ratio (SNR) were determined and compared for both phantom AC methods. Reconstruction parameters of an iterative 3D OP-OSEM reconstruction were optimized for highest lesion SNR in NEMA IQ phantom imaging.

**Results:**

Using a CT-based NEMA IQ phantom μ-map on the PET/MR system is straightforward and allowed performing accurate NEMA IQ measurements on the hybrid system. MR-based AC was determined to be insufficient for PET quantification in the tested NEMA IQ phantom because only photon attenuation caused by the MR-visible phantom filling but not the phantom housing is considered. Using the suggested CT-based AC, the highest SNR in this phantom experiment for small lesions (<= 13 mm) was obtained with 3 iterations, 21 subsets and 4 mm Gaussian filtering.

**Conclusion:**

This study suggests CT-based AC for the NEMA IQ phantom when performing PET NEMA IQ measurements on an integrated PET/MR hybrid system. The superiority of CT-based AC for this phantom is demonstrated by comparison to measurements using MR-based AC. Furthermore, optimized PET image reconstruction parameters are provided for the highest lesion SNR in NEMA IQ phantom measurements.

## Background

In diagnostic medical imaging the performance of imaging modalities needs to be tested and evaluated on a regular basis in order to ensure correct functionality and optimal image quality. Systems are analyzed by a variety of methods [1, 2]. For PET scanners in particular, the National Electrical Manufacturers Association (NEMA) has defined a standard to assess the performance of the tomographic system [3]. Such image quality control measurements also need to be conducted in PET/MR hybrid imaging for PET performance measurements when introducing a new system [1, 2] or, on a regular basis, when monitoring quality of a specific PET system over time. Comparability in clinical studies evaluating PET/CT and PET/MR imaging performance in patient studies also relies on NEMA IQ phantom measurements [4, 5]. Furthermore, accurate NEMA IQ phantom measurements are a precondition for studies investigating the attenuating influence of new hardware components such as radiofrequency (RF) coils [6, 7] and radio therapy planning equipment [8] that are designed for use in PET/MR systems. Dose optimization studies that have been reported for PET/MR hybrid imaging also rely on NEMA IQ phantom measurements [9]. All these studies have in common that they build on accurate methods for attenuation correction (AC) of the phantoms involved.

**Fig. 1:**
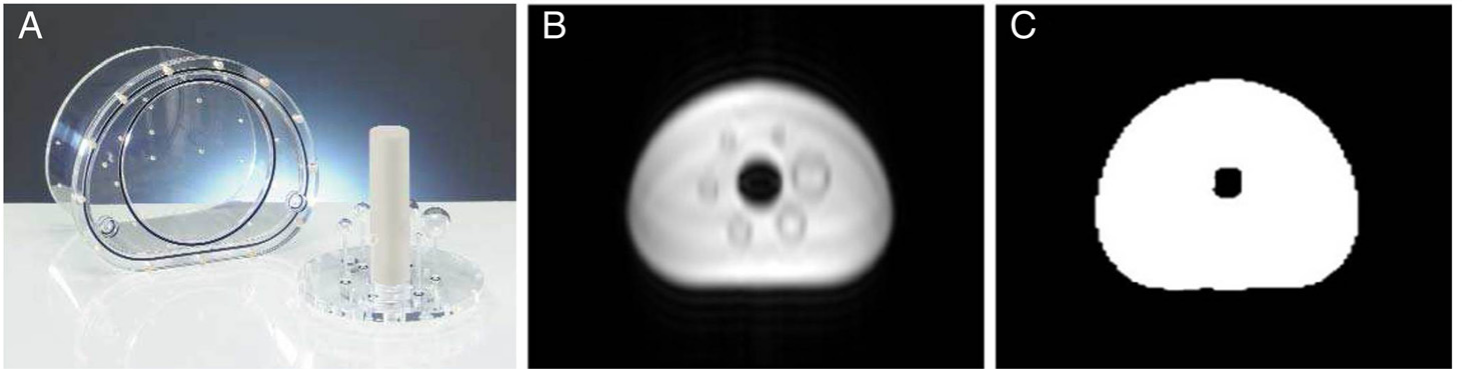
**a** The NEMA IQ phantom. **b** MR imaging (in-phase image) of the NEMA IQ phantom using the MR AC Dixon VIBE sequence showing only fluid phantom filling (water). The phantom housing (Plexiglas) is not visible in MR imaging and thus, also not considered in the MR-based μ-map of the phantom (**c**). Note that the glass spheres are displayed as signal voids (dark rims in **b**) that are segmented as water in the MR-based μ-map (**c**)

To obtain quantitative PET images that can be used to determine the scanner performance parameters, the acquired PET data need to be corrected for attenuation of the photons caused by the scanned object as well as by the attenuating hardware components of the system [10]. In PET/CT imaging, information about the attenuating characteristics of the scanned object is derived from the CT scan itself. In PET/MR patient imaging, MR-based attenuation correction (AC) methods are applied to correct for attenuation caused by human tissue [11], however, the applicability in phantom imaging has not yet been quantified. Current MR-based AC only considers the fluid phantom filling as it provides sufficient MR signal, but it does not correct for plastic or glass materials commonly used in PET phantom housings, as these cannot be reliably detected in standard MR imaging (Fig. 1).

In this study, therefore, an alternative strategy for performing NEMA IQ phantom measurements is evaluated. The use of CT-based AC maps and templates for attenuation correction of hardware components such as the PET/MR system patient table and various stationary and mechanically rigid radiofrequency (RF) coils can be considered as the current standard approach for hardware component AC in combined PET/MR systems across all vendors. Consequently, this study proposes to use a pre-acquired CT-based template μ-map of the filled NEMA IQ phantom in conjunction with a phantom holder and to install this template μ-map on the PET/MR system to be used for AC whenever NEMA IQ phantom experiments are performed.

This study investigates the standard NEMA IQ test utilizing CT-based AC for the Biograph mMR PET/MR hybrid system (Siemens Healthcare, Erlangen, Germany). Contrast recovery, background variability and signal-to-noise ratio are determined as a function of different reconstruction parameters. In comparison, the effect of MR-based AC is evaluated and the impact on the image quality parameters assessed.

## Methods

### PET/MR hybrid scanner

All phantom experiments were performed on an integrated PET/MR whole-body hybrid system (Biograph mMR, Siemens AG) [2].

The MR component of the hybrid system consists of a 3.0 Tesla static magnetic field, a radiofrequency (RF) transmit body coil and a gradient coil system which provides a maximum amplitude of 45 mT/m and a maximum slew rate of 200 T/m/s.

The PET component is comprised of 8 detector rings, each consisting of 56 detector blocks. One detector block consists of 8x8 lutetium oxyorthosilicate (LSO) scintillator crystal elements connected to 3 x 3 avalanche photodiodes (APDs) [12].

### NEMA image quality phantom

According to the NEMA NU 2–2007 standard [3], image quality parameters of PET scanners are obtained by measuring a specific International Electrotechnical Commission (IEC) 61675–1 emission phantom [13] (NEMA image quality phantom) (Fig. 1a, PTW, Freiburg, Germany). This image quality phantom mimics the shape of an upper human body and is built of acrylic glass material. It comprises 6 hollow glass spheres (inner diameters 37, 28, 22, 17, 13, and 10 mm) which can be inserted into the large phantom compartment. Additionally a cylindrical insert containing styrofoam with an average density of 0.3 ± 0.1 g ml^−1^ (simulates patient lung tissue [3] (μ_lung-insert_ ~ 0.026 cm^−1^) and is positioned in the center of the phantom. The inner volume may vary between NEMA IQ phantoms. The volume of the tested phantom (PTW, Freiburg, Germany, Fig. 1a) was measured to be 9.5 L ± 1 % when the spheres and lung cylinder are inserted. The phantom housing has a thickness of approximately 3 mm along the phantom body, and 10 mm (in few parts 20 mm) at the lids at both ends of the phantom. The glass material (μ ~ 0.118 cm^−1^) of the spheres has a thickness of around 1 mm.

### Phantom measurement setup

When NEMA IQ measurements with a CT-based phantom template μ-map are performed, the phantom needs to be placed at a pre-defined position in the PET field of view (FOV) with a known reference to the patient table, to ensure alignment between the μ-map and the position of the phantom.

**Fig. 2:**
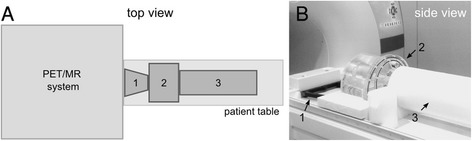
The NEMA IQ phantom imaging setup as performed in this study. The schematic drawing in (**a**) and the image in (**b**) show the spacer (1), the NEMA IQ phantom (2), and the scatter phantom (3) arranged on top of the PET/MR system patient table. The spacer (1) ensures a predefined and reproducible position of the phantom (2) on the patient table

To guarantee a reproducible phantom placement, a defined set of phantom holders were used (Fig. 2). A spacer is positioned adjacent to the RF head coil connection port on the patient table in order to create a defined and reproducible distance of the NEMA IQ phantom to the stronger photon-attenuating RF coil port. The NEMA IQ phantom is placed next to the spacer on a foam block, which was designed in order to align the phantom at a pre-defined patient table position. As required by the NEMA standard, a scatter phantom (a 70 cm long plastic cylinder with an activated line source) is positioned contiguously to the NEMA IQ phantom to generate scattered and random coincidences from outside the FOV, such as in a patient examination (Fig. 2).

### PET phantom preparation and data acquisition

The background volume of the NEMA IQ phantom and the four smallest spheres were filled with ^18^ F-FDG mixed with pure water using a 4:1 sphere-to-background activity concentration ratio, as specified by NEMA [3]. The initial tracer activity concentration was specifically calibrated to the start of the measurement: 5.3 kBq/ml ± 1 % in the phantom background and 21.2 kBq/ml ± 5 % in the four smallest spheres. The two largest spheres were filled with water only. The non-radioactive cylindrical insert simulating lung tissue was placed in the center of the phantom. The line source contained in the large scatter phantom was injected with 110 MBq of ^18^ F-FDG [3].

The defined measurement time by the NEMA NU 2–2007 standard is dependent on the axial imaging distance of the PET system and amounted to 12 min in one bed position for the Biograph mMR. This imaging time resulted from the specification of NEMA NU 2–2007 that 100 cm of axial imaging distance shall be covered in 60 min. This time was adapted in the NEMA 2012 standard to 30 min.

The PET images were reconstructed using the 3D ordinary Poisson ordered-subset-expectation-maximization (OP-OSEM) reconstruction algorithm [14] as implemented in the system.

### NEMA image quality parameters

**Fig. 3:**
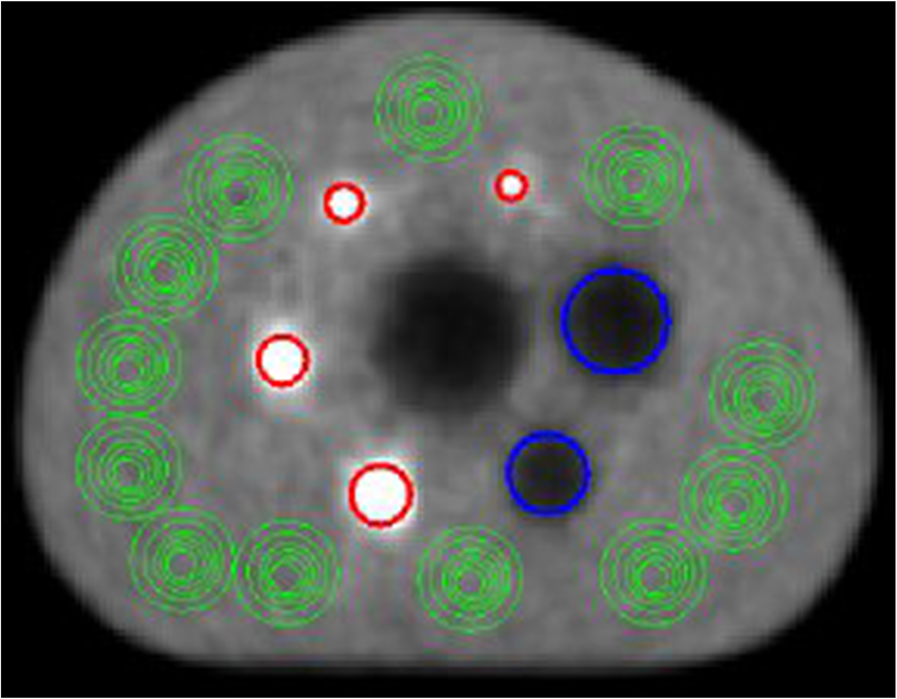
Position of the regions of interest (ROIs) placed over the active spheres (*red*), nonradioactive spheres (*blue*) and the phantom background (green) of the reconstructed PET images, which are used for analysis

According to the NEMA NU 2–2007 protocol, PET image quality is analyzed by means of two parameters: contrast recovery and background variability [3, 15]. These parameters are calculated by evaluation of various regions of interest (ROIs) in the transverse image slice that contains the centers of the spheres, as well as in adjacent slices (Fig. 3), as defined by the NEMA standard. The ROIs are defined on the attenuation corrected PET image and are

drawn over the spheres as well as in the background region as is illustrated in Fig. 3. In this study the evaluation of the ROIs was performed with the open-source image analysis tool ImageJ (Fiji).

The percentage contrast recovery (in an ideal case = 100 %) is determined for each hot sphere *j* by:

$$ {Q}_{H,j}=\frac{\raisebox{1ex}{${C}_{H,j}$}\!\left/ \!\raisebox{-1ex}{${C}_{B,j}$}\right. - 1}{\raisebox{1ex}{${a}_H$}\!\left/ \!\raisebox{-1ex}{${a}_B$}\right. - 1}*100\ \left[\%\right] $$

C_H,j_ = average counts in the ROI for sphere j

C_B,j_ = average counts in the background ROI for sphere j

a_H_ = activity concentration in the hot spheres

a_B_ = activity concentration in the background

For each nonradioactive sphere *j* the percentage contrast recovery *Q*_*C,j*_ is given by:

$$ {Q}_{C,j} = \left(1 - \frac{C_{C,j}}{C_{B,j}}\right)*100\ \left[\%\right] $$

*C*_*C,j*_ = average counts in the ROI for sphere *j*

*C*_*B,j*_ = average of all background ROI counts for sphere *j*

In order to determine the percentage background variability *N*_*j*_ as a measure for the image noise for sphere *j* (in an ideal case = 0 %), the following equation is used:

$$ {N}_j = \frac{S{D}_j}{C_{B,j}}*100\ \left[\%\right] $$

*SD*_*j*_ = standard deviation of the background ROI counts for sphere *j*

*C*_*B,j*_ = average of all background ROI counts for sphere *j*

### NEMA IQ measurement with MR-based AC

To evaluate the effect of neglecting acrylic glass and glass material in the attenuation correction of the NEMA IQ phantom emission data, reconstructions with an MR-based μ-map containing only the fluid phantom filling were performed and NEMA IQ parameters were calculated. Linear attenuation coefficients of water were assigned to the whole phantom content including phantom liquid and glass inserts, despite of higher attenuation of the glass material of the spheres. For reconstruction, the above-mentioned OP-OSEM algorithm was used with 3 iterations, 21 subsets, 172 matrix size, and 4 mm Gaussian post-smoothing filter. MR-based AC was performed by a 3D Dixon VIBE-based approach [11] with the following imaging sequence parameters: TR: 4.07 ms, TE in-phase: 2.46 ms, TE opposed-phase: 1.23 ms, flip angle: 10°, slice thickness: 3.12 mm, field of view: 500 mm x 328 mm and matrix size: 128 x 84. The system’s built-in RF transmit body coil was used for RF transmission and signal reception. All other RF coils were removed from the patient table to avoid additional PET photon attenuation [16].

**Fig. 4:**
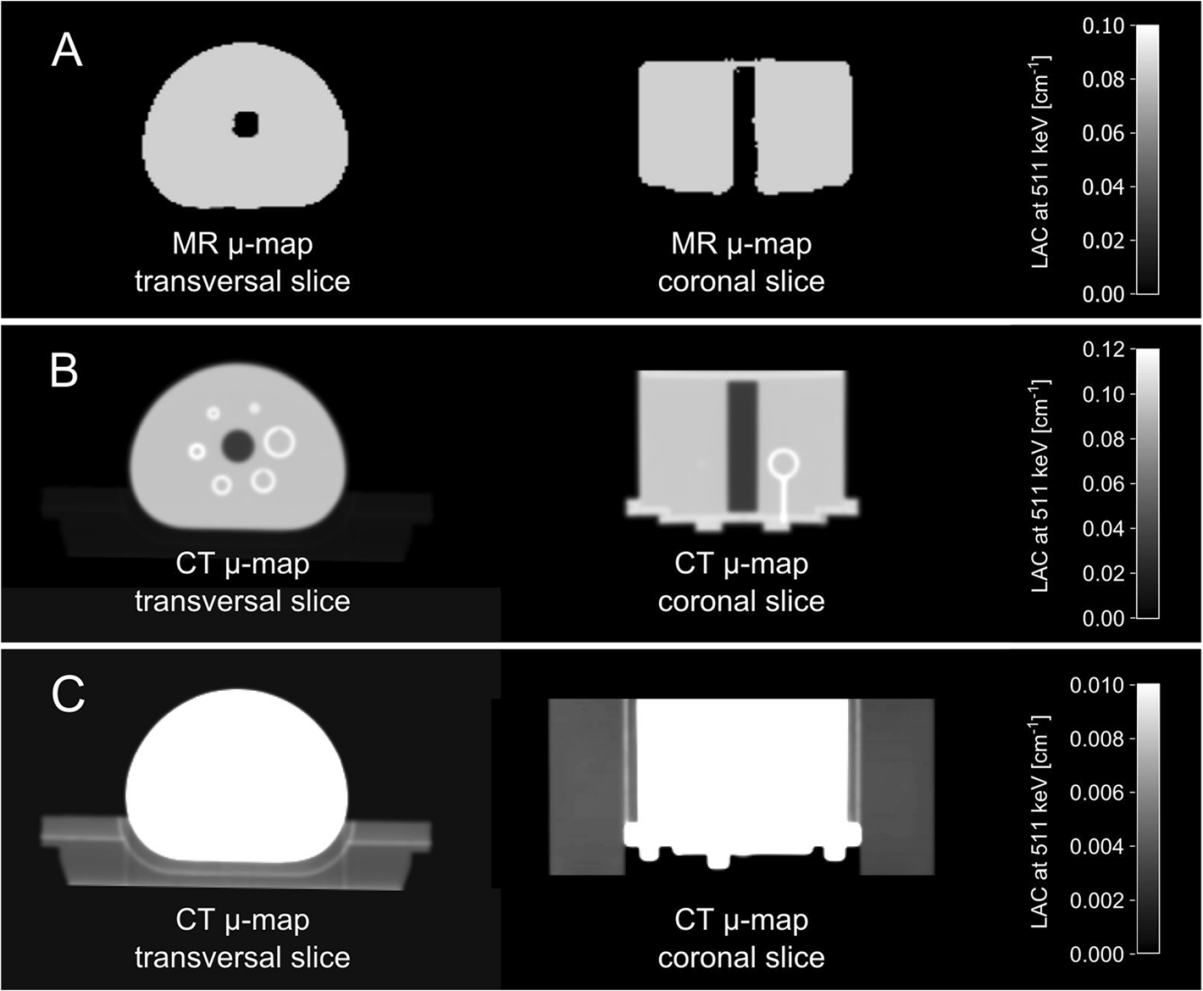
**a** MR-based μ-map in transversal and coronal orientation only contains discrete attenuation values for water and air, and therefore only corrects for photon attenuation caused by the water content of the phantom and not by the phantom housing materials, as these materials cannot be detected with standard MR imaging. **b**, **c** CT-based μ-map contains continuous attenuation values including μ-values for the phantom housing, glass spheres, and styrofoam block used as phantom holder (displayed in **c**). **b** and **c** visualize the same content, however windowing properties were adjusted individually in order to visualize either the phantom content (**b**) or the styrofoam holder, on which the phantom is placed (**c**)

The assigned μ-values were restricted to two μ-values for water and air for the performed phantom measurements. Attenuation caused by the styrofoam material in the lung insert was not accounted for. In general, imaging the NEMA IQ phantom filled with pure water on a 3.0 Tesla MR system leads to artifacts and signal inhomogeneities due to standing-RF-wave phenomena and T1 effects, thus affecting MR-based AC of the NEMA phantom in PET/MR hybrid imaging (as shown in [17]). Manually reducing the initial voltage of the RF transmitter adjustment algorithm led to a lower adjusted transmitter voltage of 92.7 Volts, instead of the default value for patient imaging (~300 V), and resulted in fairly homogeneous μ-maps of the NEMA IQ phantom filling (Fig. 1c, Fig. 4a) [17]. Alternatively the addition of substances (e.g. NiSO_4_) could be considered that decrease the T1 relaxation time of water and as a consequence decrease the mentioned image artefacts, as discussed in [17]. However, this was not further evaluated in the present study.

### NEMA IQ measurement with CT-based AC

The CT image of the phantom, which was used to generate the CT-based template μ-map (Fig. 4b and c), was acquired on a 128-slice PET/CT system (Biograph 128, Siemens AG) with the following parameters: 500 effective mAs and 140 kVp. Images were reconstructed with 0.6 mm slice thickness, 512 x 512 image matrix size and a B30f convolution kernel. Following a 2 mm Gaussian filtering and bilinear scaling of the CT Hounsfield units to linear attenuation coefficients at the PET energy level of 511 keV [18], the CT-based μ-map was installed on the PET/MR system in order to be used for AC whenever NEMA IQ measurements are performed.

The phantom holder minimizes misalignment between the actual phantom position and the CT-based template AC map of the phantom as much as possible. In case of remaining slight misplacement between CT-based phantom template and actual phantom position, an image-based registration can be performed. A practical use of a CT-AC method as proposed in this study by potential future PET/MR users will require the registration to be performed based solely on the images available. Thus the registration in this study was also performed by means of the available information from the PET and CT images in order to simulate a realistic case. This was performed by using the inherent landmarks of the phantom such as the glass spheres, the lung insert and the phantom outer boundary that function as orientation when performing manual image registration.

For the PET measurements utilizing CT-based AC, reconstruction parameters were set to 3D OP-OSEM with 3 iterations, 21 subsets, 172 matrix size and 4 mm Gaussian filtering, as also used with MR-based AC.

### Optimization of PET reconstruction parameters in phantom images

PET performance parameters, as well as the visibility of lesions in phantom and patient images, in general strongly depend on the applied reconstruction algorithm parameters. To investigate this effect in the specific context of NEMA IQ phantom imaging particularly for the Biograph mMR system and its PET detector geometry and reconstruction algorithm, different reconstructions of the same PET phantom data acquisition using CT-based attenuation correction were evaluated. Hereby the number of iterations (1–5 iterations), the image matrix size (172 x 172, 344 x 344 matrix) and the Gaussian filter (2 mm, 4 mm) were varied and the resulting impact on the image quality was investigated, as has been discussed often in the literature and is described e.g. in [19]. The number of subsets was kept constant at 21 as this is the default setting on the scanner console. To determine the optimal reconstruction parameters for best NEMA IQ phantom image quality with high lesion contrast and low background noise, the signal-to-noise ratio (SNR) was selected as a representative image quality measure and was calculated for each active sphere and for all listed reconstruction parameter combinations. The SNR for sphere *j* was determined, as described in the literature when performing similar evaluations for PET/CT imaging [20, 21], according to the following equation:

$$ SN{R}_j = \frac{Signa{l}_j- Background}{\sigma_B\ } $$

*Signal*_*j*_ *=* average counts in the ROI for sphere *j*

*Background =* average counts in a ROI placed in a uniform area outside the

spheres

*σ*_*B*_ *=* standard deviation of the background ROI counts, corresponding to noise in the image

## Results

### NEMA IQ measurement with MR-based AC

The resulting NEMA IQ parameters utilizing MR-based AC are listed in Table 1. Regarding the four smallest spheres filled with tracer activity (“radioactive spheres”) the performance values using MR-based AC are lower than the expected values for the underlying PET component when comparing as a general orientation to results reported in the literature for the Biograph mMR system [2]. The contrast recovery values for the two largest spheres filled with non-radioactive water only (“non-radioactive spheres”) (Table 1) show higher values when compared to the results reported in the literature for the Biograph mMR system [2].

**Table 1:** Contrast recovery and background variability parameters when applying MR-based AC

Sphere size [mm]	Radioactive / Non-radioactive	^a^Contrast recovery [%]	^a^Background variability [%]
10	Radioactive	16.8 ± 1.9	4.8 ± 0.8
13	Radioactive	31.7 ± 2.9	4.0 ± 0.4
17	Radioactive	52.7 ± 3.0	3.6 ± 0.3
22	Radioactive	64.8 ± 4.1	3.4 ± 0.4
28	Non-radioactive	68.9 ± 1.5	3.2 ± 0.4
37	Non-radioactive	76.1 ± 0.6	3.1 ± 0.3

^a^ The values represent the mean values and standard deviation of four independent measurements for the reconstruction: 3 iterations, 21 subsets, 172 matrix and 4 mm Gaussian filtering

### NEMA IQ measurement with CT-based AC

Table 2 presents the resulting NEMA IQ parameters when applying CT-based AC of four independent measurements with separate fillings on four different days. In comparison to values obtained when applying MR-based AC (Table 1), contrast recovery was, for instance for the smallest sphere, almost approximately twice as high when using CT-based AC. The larger the size of the spheres, the smaller the deviation in contrast recovery to MR-based AC becomes. The results using CT-based AC are in the same range as results obtained in the literature [2].

**Table 2:** Contrast recovery and background variability parameters when applying CT-based AC

Sphere size [mm]	Radioactive / Non-radioactive	^a^Contrast recovery [%]	^a^Background variability [%]
10	Radioactive	30.5 ± 1.3	3.8 ± 0.8
13	Radioactive	50.5 ± 2.4	3.0 ± 0.5
17	Radioactive	72.9 ± 1.9	2.5 ± 0.2
22	Radioactive	74.5 ± 3.3	2.3 ± 0.2
28	Non-radioactive	56.6 ± 2.3	2.0 ± 0.2
37	Non-radioactive	64.8 ± 0.9	1.8 ± 0.1

^a^ The values represent the mean values and standard deviation of four independent measurements for the reconstruction: 3 iterations, 21 subsets, 172 matrix and 4 mm Gaussian filtering

### Optimization of PET reconstruction parameters of 3D OP-OSEM

**Fig. 5:**
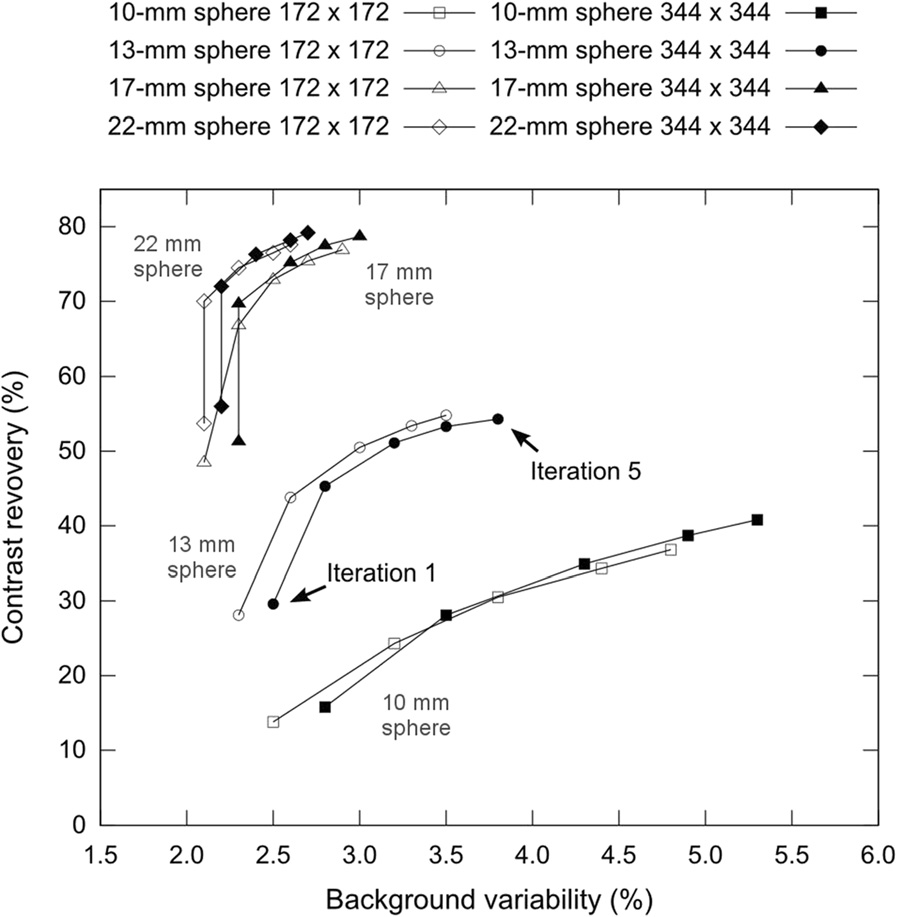
Contrast recovery vs. background variability using CT-based AC shown for the four smallest spheres with 172x172 matrix size (hollow symbols) and 344x344 matrix size (solid symbols) and 4 mm Gaussian filter. The values from left to right represent the results for an increasing number of iterations (1–5) of the reconstruction algorithm

The influence of varying reconstruction parameters is demonstrated for the four smallest spheres in Table 3 and plotted in Fig. 5. It can be determined that contrast recovery increases with an increasing number of iterations at the cost of higher background variability, which is essentially noise.

**Table 3:** Contrast recovery and background variability values in % for each active sphere for different reconstruction parameters of the 3D OP-OSEM reconstruction algorithm when using CT-based AC

	^a^Contrast recovery [%] │ ^a^Background variability [%]							
	Sphere 10 mm		Sphere 13 mm		Sphere 17 mm		Sphere 22 mm	
Iteration	172 x 172	344 x 344	172 x 172	344 x 344	172 x 172	344 x 344	172 x 172	344 x 344
1	13.8 │ 2.5	15.8 │ 2.8	28.1 │ 2.3	29.6 │ 2.5	48.5 │ 2.1	51.3 │ 2.3	53.7 │ 2.1	56.0 │ 2.2
2	24.3 │ 3.2	28.1 │ 3.5	43.8 │ 2.6	45.3 │ 2.8	66.8 │ 2.3	69.7 │ 2.3	70.0 │ 1.9	72.0 │ 2.2
3	30.5 │ 3.8	35.0 │ 4.3	50.5 │ 3.0	51.1 │ 3.2	72.9 │ 2.5	75.2 │ 2.6	74.5 │ 2.3	76.3 │ 2.4
4	34.3 │ 4.4	38.7 │ 4.9	53.4 │ 3.3	53.3 │ 3.5	75.4 │ 2.7	77.5 │ 2.8	76.5 │ 2.5	78.2 │ 2.6
5	36.8 │ 4.8	40.8 │ 5.3	54.8 │ 3.5	54.3 │ 3.8	76.9 │ 2.9	78.7 │ 3.0	77.6 │ 2.6	79.2 │ 2.7

^a^4 mm Gaussian filtering and 21 subsets were kept constant for all reconstructions. The values represent the mean of four independent measurements

**Fig. 6:**
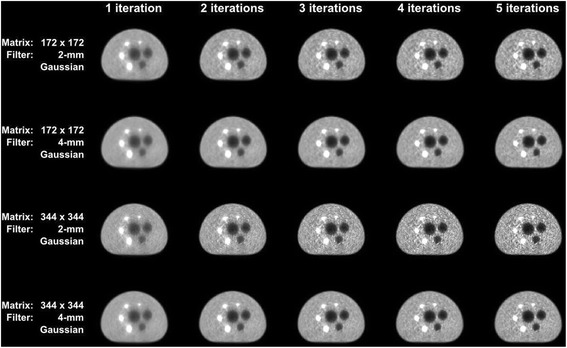
Images of the first five iterations reconstructed with 3D OP-OSEM for different matrix and Gaussian filter sizes and using CT-based AC. Note: increasing the number of iterations is associated with increasing lesion sharpness while also increasing background noise. This also applies when increasing the image matrix size from 172 x 172 to 344 x 344

When comparing two different matrix sizes (344 x 344 and 172 x 172), it can be observed that with higher image matrix the contrast increases but at the same time it causes an increase in noise. These effects can also be perceived in the phantom images in Fig. 6.

**Fig. 7:**
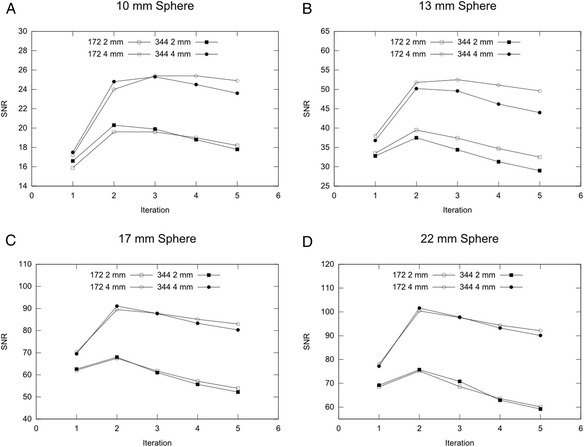
The signal-to-noise ratio (SNR) calculated for different reconstruction parameter combinations for the four active spheres: 10 mm (**a**), 13 mm (**b**), 17 mm (**c**) and 22 mm (**d**). The values represent the mean of four independent NEMA IQ measurements using CT-based AC

The mean SNR of four independent measurements as a function of iterations for all investigated reconstruction parameter combinations is plotted for each of the four smallest spheres in Fig. 7. The best choice of matrix size, Gaussian filter size and number of iterations can be determined from the peak SNR value for each sphere in the graphs. As can be seen, highest SNR is achieved for all spheres using a 4 mm Gaussian filter. For the two larger spheres (17 and 22 mm) 2 iterations led to highest SNR, whereas for the two smallest spheres (10 and 13 mm) the peak SNR was achieved at 3 iterations. No significant difference in resulting SNR values can be observed when comparing 344 x 344 to 172 x 172 matrix size. Therefore the matrix should be selected with respect to the individual application.

## Discussion

In this study, a strategy for using CT-based phantom attenuation correction in the context of NEMA IQ measurements in PET/MR hybrid imaging has been evaluated. The proposed strategy features a pre-acquired CT-based 3D attenuation template of the NEMA IQ phantom in conjunction with a phantom holder providing exact and reproducible repositioning of the phantom on the systems patient table. This strategy for phantom AC is straightforward and in line with currently implemented AC methods for hardware component AC such as RF coils on the available sequential and integrated PET/MR systems. Respective image quality and activity quantification parameters were systematically investigated using MR-based and CT-based AC of the NEMA IQ phantom. Furthermore, reconstruction parameters for NEMA IQ phantom measurements were optimized for the Biograph mMR system by evaluating image quality parameters using the CT-based approach.

It was demonstrated that the tested MR-based AC leads to insufficient quantification results and to degraded image quality as compared to CT-based AC for the evaluated NEMA IQ phantom. Specifically, the activity quantification values for the four “radioactive spheres” were lower than activity values reported in the literature for NEMA IQ measurements on the Biograph mMR system [2]. The low values indicate insufficient AC of the phantom, which is associated with the fact that MR-based AC only considers the fluid phantom filling but neglects the attenuating phantom housing made from acrylic glass and glass components. On the other hand, the contrast recovery values for the two “non-radioactive spheres” show higher values when compared to the values reported in the literature [2]. Because these spheres are filled with non-radioactive water only, the corresponding contrast recovery values are highest when no counts are detected from inside these spheres. Applying an insufficient attenuation correction, such as the MR-based AC, leads to less counts being detected from inside the non-radioactive spheres resulting in a higher contrast recovery value than when using CT-based AC. Based on the resulting contrast recovery parameters for radioactive and non-radioactive spheres it can be concluded that MR-based attenuation correction performed with the current AC sequence is inadequate to determine representative PET image quality parameters in the tested NEMA IQ phantom.

The proposed method, using pre-acquired CT-based NEMA IQ phantom attenuation templates instead of MR-based AC, takes the complete phantom into account, including the fluid filling, phantom housing, lung insert and foam phantom holder. Using this approach leads to NEMA PET image quality parameters similar to values reported in the literature [2]. The CT-based approach can thus be considered adequate for representing the performance characteristics of the Biograph mMR PET component.

The suggested CT-based AC requires the NEMA IQ phantom to be located at a known position on the patient table during the measurements. This was achieved by a foam block that carries the NEMA IQ phantom and fits the shape of the systems patient table. To allow for exact and reproducible positioning of the foam block in z-direction on the patient table, a spacer was used, which is already part of the system. The proposed approach can theoretically be implemented on any current PET/MR system (tri-modality, sequential or integrated) as a practical method to perform PET NEMA image quality measurements.

In theory, other methods are conceivable to obtain improved MR-based AC of the NEMA IQ phantom. In principle this requires that the phantom fluid is displayed in homogeneous manner across the entire phantom [17]. Another precondition is that the phantom housing made e.g. of Plexiglas is also considered in MR-based AC. This may be achieved by using MR imaging sequences capable of displaying structures made of plastic, e.g. ultrashort echo time (UTE) sequences. Alternatively, the virtual addition of a defined rim of attenuating pixels to the outer borders of the phantom fluid visible in MR imaging may provide a method for consideration of the phantom housing in MR-based AC. Additionally, a 511 keV transmission scan of the phantom may also be a possibility to generate a phantom AC map. However, a very long scan would be required in order to limit the noise properties, which are much higher in comparison to CT, and achieve acceptable spatial resolution. In addition, since the advent of combined PET/CT scanners access to PET only scanners with transmission sources is very limited. In the present study we have focused on the CT-based AC strategy since it is straightforward, accurate, and can be implemented on current PET/MR systems in the same manner hardware component AC is performed for RF coils.

A goal of this study was to assess a solution to be able to perform NEMA PET image quality evaluations on a PET/MR system as closely to the NEMA NU 2–2007 standard as possible. NEMA NU 2–2007 specifies that the IQ phantom shall be positioned in the isocenter of the PET FOV. Since the patient table does not accommodate this vertical phantom position, a suggested phantom holder ensures the required positioning in a reproducible manner. It reduces misplacement of the phantom in all three spatial dimensions as much as possible and consequently, the position of the phantom during a NEMA measurement matches the position of the CT-based template AC map of the phantom. In case of remaining slight misalignment, manual post-registration and repeated reconstruction is possible. In this case the phantom μ-map was registered manually to the non-attenuation corrected PET data. Possibly, manual or automatic registration to MR data may be another alternative, however, due to suboptimal image quality of the MR data acquired with the RF body coil, this was not performed for the presented data.

For the 3D OP-OSEM reconstruction in this NEMA IQ phantom study, an optimized lesion SNR was achieved by reconstructing with 3 iterations, 21 subsets and 4 mm Gaussian filtering. The image matrix size should be selected according to the application. In theory every lesion in patient data sets has a different optimal reconstruction parameter setting to obtain the highest individual lesion SNR. Therefore, the parameter optimization in this study can be considered only a general guideline when performing PET patient measurements on the Biograph mMR hybrid system.

## Conclusion

This study validates NEMA (NU 2–2007) PET image quality performance measurements by applying CT-based attenuation correction for an integrated PET/MR hybrid system. The necessity and superiority of CT-based NEMA IQ phantom AC is demonstrated by a comparison to results using MR-based AC. Furthermore, optimized image reconstruction parameters are provided for highest lesion SNR in the context of NEMA IQ phantom measurements on the Biograph mMR system. The results of this study can thus be seen as an important step towards standardization and image quality control of PET measurements in the context of PET/MR hybrid imaging.
